# The Role of Age-Friendly Environments on Quality of Life among Thai Older Adults

**DOI:** 10.3390/ijerph14030282

**Published:** 2017-03-09

**Authors:** Sariyamon Tiraphat, Karl Peltzer, Kriengsak Thamma-Aphiphol, Kawinarat Suthisukon

**Affiliations:** 1 ASEAN Institute for Health Development, Mahidol University, Salaya, Phutthamonthon, Nakhon Pathom 73170, Thailand; sariyamon.tir@mahidol.ac.th (S.T.); kriengsak.sue@mahidol.ac.th (K.T.-A.); kawinarat.sut@mahidol.ac.th (K.S.); 2 Department of Psychology, University of the Free State, Bloemfontein 9300, South Africa; 3 HIV/AIDS/STIs and TB (HAST), Human Sciences Research Council, Pretoria 0001, South Africa

**Keywords:** age-friendly environments, quality of life, physical environment, social environment, older adults, Thailand

## Abstract

Studies on the significance of age-friendly environments towards quality of life among older adults have been limited. This study aimed to examine the association between age-friendly environments and quality of life among Thai older adults. Cross-sectional interview survey data were collected from 4183 older adults (≥60 years) using multistage stratified systematic sampling from all four regions in Thailand. The outcome variable was the World Health Organization Quality of Life (WHOQOL-BREF) scale, while independent variables included sociodemographic factors, having a health problem, and neighbourhood age-friendly environment variables. In multivariable logistic regression, significant age-friendly environments predictors of quality of life included walkable neighbourhood, neighbourhood aesthetics, neighbourhood service accessibility, neighbourhood criminal safety, neighbourhood social trust, neighbourhood social support, and neighbourhood social cohesion. The present study confirms the important role of age-friendly neighbourhoods in terms of physical and social environments towards the quality of life of older adults.

## 1. Introduction

Global life expectancy is increasing each year. The World Health Organization (WHO) reported that “average life expectancy at birth in 1955 was just 48 years; in 1995 it was 65 years; in 2025 it will reach 73 years” [1]. The longer life expectancy causes a rapid increase in the number of older adults (60 years and older), doubling from 600 million in 2017 to 1.2 billion in 2025 [2]. Due to a longer life expectancy, maintaining a better quality of life is challenging.

The WHO indicated that not only genes and personal characteristics, but also physical and social environments can play an important role in determining people’s health and well-being throughout people’s life course [3]. In order to prepare for demographic ageing, the WHO developed guidelines on age-friendly environments, including eight domains: “Areas of urban living: outdoor spaces and buildings; transportation; housing; social participation; respect and social inclusion; civic participation and employment; communication and information; and community support and health services.” [4]. Age-friendly physical and social environments are designed to encourage and support active ageing by enhancing opportunities for health, participation and security so as to improve the quality of life of the ageing population [2,4].

Previous studies found that the following age-friendly environment factors may be associated with better quality of life, well-being, life satisfaction, or self-rated health among older adults: social environment such as social support from family, friends and neighbours [5,6,7,8], neighbourhood social cohesion [8,9,10,11,12,13], social trust [14,15], an aesthetic neighbourhood or pleasant physical environment [11,16,17,18], a walkable neighbourhood [19,20], neighbourhood service accessibility [5,21,22], perceived neighbourhood security and safety [23,24,25], and neighbourhood crime safety [26,27]. There is a lack of studies in Southeast Asia, including Thailand, investigating age-friendly environments and how these impact quality of life among older adults.

Therefore, this study aimed to examine the association between age-friendly environments and quality of life among older adults in Thailand. Evidence of a significant relationship between age-friendly environments and quality of life among older adults will support health and social authorities to increase efforts in building age-friendly environments.

## 2. Methodology

### 2.1. Description of Survey and Study Population

This research design was a cross-sectional interview household survey using a multistage, stratified sampling procedure collecting data in all four regions of Thailand and the Bangkok Metropolitan Administration (BMA). We calculated the sample size using the total number of the Thai older population aged 60 years and older as 10,014,705 obtained from the National Statistical Office (NSO) of Thailand [28]. The sample size for this study was calculated by using Krejcie and Morgan formula, with the standard population proportion of 50%, and degree of accuracy at 0.015. After adding about 10% of the sample size to information loss due to incomplete data or withdrawal of participants, the final sample size was 4316 cases of the older population. According to the NSO, among the whole population aged 60 years and older, 9% of older adults live in the BMA, 26% in the central, 21% in the northern, 32% in the northeastern, and 12% live in the southern region. Therefore, a representative sample of the older adult population in Thailand included the following proportions for each region: 404 from the BMA, 1101 from the central, 914 from the northern, 1379 from the northeastern, and 518 from the southern region. At the first sampling stage, two provinces were randomly selected from all 25 provinces in the central region, two from nine provinces in the northern region, three from 20 provinces in the northeastern region, and four from 14 provinces in the southern region. At the second stage, two districts were randomly selected from 7 to 15 districts in each province, including the BMA. At the third step, in each district, villages were selected based on urban and rural areas. At the final step, every person 60 years of age and older living in the randomly selected households in the study area was eligible for the study. Finally, from all the eligible respondents in a household, one was randomly selected for the interview.

After excluding the observations with missing data, 4183 observations remained in the sample. This research project received ethical approval from the Research Ethics Committee of the Faculty of Social Sciences and Humanities, Mahidol University (Certificate of Approval No. 2015/151.2204). Informed consent was obtained from all study participants.

### 2.2. Measures

Quality of life was assessed with the World Health Organization Quality of Life (WHOQOL-BREF) questionnaire-Thai version [29], which has been validated with an older adult population in Thailand showing a good validity of 0.65 [28]. The WHOQOL-BREF-THAI is composed of 26 items for evaluating four main dimensions of quality of life including physical, psychological, social relationships and environment domains. Each individual item of the WHOQOL-BREF is scored from 1 to 5 on a response ordinal scale, with higher scores indicating a higher quality of life. Three items of the questionnaire must be reversed before scoring. In this study, the sum score ranged from 30 to 130. Finally, we dichotomized the quality of life as poor/normal and good quality of life, using a standard cutting point criteria as below 96 for poor/normal quality of life and 96 or above for good quality of life [29]. Internal consistency for the WHOQOL-BREF in this study sample was α = 0.93.

Age-friendly environments were assessed with two scales: (1) the 23-item “Neighbourhood Environment Walkability Scale-Abbreviated” (NEWS-A) [30], adapted Thai version [31]; and (2) a nine-item neighbourhood social environment index [19].

(1) The NEWS-A included in this study consisted of six sub-scales, including: (A) service accessibility (four items, e.g., “It is not too far for you to walk from your home to stores such as grocery, drug store, fresh fruit and vegetable market, or supermarket.”) (Cronbach α = 0.83); (B) street connectivity (three items, e.g., “In your neighbourhood, there are many ways to choose to go from one place to another.”) (Cronbach α = 0.73); (C) infrastructure and safety for walking and cycling (six items, e.g., “The sidewalk in your neighbourhood is mostly along the road.”) (Cronbach α = 0.79); (D) aesthetics (four items, e.g., “In your neighbourhood, it is generally free from litter, swamp, and other debris.”) (Cronbach α = 0.76); (E) traffic hazards (three items, e.g., “In your neighbourhood there is crowded traffic nearby, making it difficult and unsatisfying to walk.”) (Cronbach α = 0.86); and (F) crime (three items, e.g., “There is a high crime rate in your neighbourhood.”) (Cronbach α = 0.87). The response options ranged from 1 (strongly disagree) to 4 (strongly agree). The subscales for traffic hazards and crime were reversed scored. Summing the mean score from each subscale, the possible scores ranged 6–24. A higher score referred to a more favorable value of the environmental characteristics or higher physical activity performance and a lower score referred to less favorable value of the environmental characteristics or lower physical activity performance. Finally, the scores of each subscale were dichotomized as low and high, using the median as the cut point.
(2) The neighbourhood social environment index consisted of three sections: (A) social trust (three items, e.g., “You generally trust your neighbour; for example, when you are not at home, you ask your neighbour to look after your property.”) (Cronbach α = 0.83); (B) social cohesion (three items, e.g., “You and your neighbours get together for activities such as community problem solving.”) (Cronbach α = 0.85); and (C) social support (three items, e.g., “Your neighbours are very pleased to help each other.”) (Cronbach α = 0.91). The instruction required participants to respond to the question “What do you think best applies to you and your neighbourhood?” The response options ranged from 1 (strongly disagree) to 5 (strongly agree). The possible scores ranged 9–45. Higher scores reflected greater satisfaction. Finally, the scores of each subscale were dichotomized as low and high, using the median as the cut point.

Sociodemographic and health variables included: age, gender, educational level, marital status, income level (1: less than 5000 Baht/month; 2: 5001–10,000 Baht/month; 3: 10,001–20,000 Baht/month; and 4: 20,001 Baht or higher per month), geolocation (1: rural area; 2: urban area; and 3: Bangkok Metropolitan Area), living arrangement (1: living alone; 2: living with others), number of years residing in current community (1: ≤10 years, 2: >10 years), and currently having a health problem such as a chronic disease, physical health problem, mental health problem and other health problem that had been diagnosed by a physician (1: no; 2: yes).

### 2.3. Data Analysis

Data were analysed using IBM Corp. IBM SPSS Statistics version 21.0 (IBM Corp, Armonk, NY, USA). Descriptive statistics was used to describe the sample. In order to examine differences in the proportion of quality of life, Pearson χ^2^ tests were applied. Multivariable logistic regression analysis was applied to investigate the predictors (socio-demographic factors, health problems, and total age-friendly environment) of good quality of life. Finally, age-friendly environmental predictors in each subscale were examined by multivariable logistic regression using the “enter method.” All predictors were checked for co-linearity, which was not of concern (Variance Inflation Factor = VIF ranging 1.2–2.4). The level of significance for all analyses was set at *p* < 0.05.

## 3. Results

### 3.1. Sample Characteristics

The total study sample included 4183 Thai older adults (60 years or more); the response rate was 95%. Almost two-thirds (64.6%) of the sample were women, more than half (54.3%) were 60 to 69 years old and were married (58.5%). Most had completed elementary school (73.9%), had an income of less than 5000 Baht per month (74.6%), and 54.4% lived in urban areas including Bangkok. Most older adults (>90%) had been living in their community for more than ten years and were living with others. The majority (76%) indicated that they had a health problem (see Table 1).

### 3.2. Descriptive Results

Approximately 50% of the participants perceived their neighbourhood as having high street connectivity, places for walking, criminal safety, and traffic safety. About 60% of the respondents perceived their neighbourhood as having high service accessibility and high aesthetics. More than 75% of the older adults agreed that their neighbourhood had high social cohesion and high social support, but only 55.6% reported high social trust in their neighbourhood. More than one-third (35.8%) of the older adults scored on the WHOQOL-BREF as having a good quality of life (see Table 2).

### 3.3. Levels of Quality of Life

The results of χ^2^ tests showed that significant socio-demographic and health factors were associated with quality of life among Thai older adults, namely higher education, higher income, younger age, being married, not having a health problem and having resided in their community longer than ten years. Gender and living arrangement were not significant factors associated with quality of life. Further, age-friendly environments were found to be significantly associated with quality of life among Thai older adults. In particular, street connectivity, places for walking, criminal safety, traffic safety, service accessibility, aesthetics, social trust, social cohesion and social support were associated with good quality of life (see Table 3).

### 3.4. Age-Friendly Environments, Socio-Demographic Factors and Health Conditions as Predictors of Quality of Life

Table 4 indicates the predictors of quality of life among the Thai older population. The results of multivariable logistic regression indicate that older adults who perceived a higher level of neighbourhood age-friendly environment were 3.79 times more likely to have a good quality of life compared to those perceiving a lower level of neighbourhood age-friendly environment. Other significant predictors of quality of life among the Thai older population include educational level, marital status, income level, age level, health problem, and number of years living in the community.

Table 5 indicates significant age-friendly environment predictors of quality of life among older adults by each age-friendly environment sub-scale. After adjusting for sociodemographic and health variables, the results of multivariable logistic regression indicate that significant age-friendly predictors of quality of life are places for walking, criminal safety, service accessibility, aesthetics, social trust, social cohesion and social support. However, the results showed that neighbourhood street connectivity and neighbourhood traffic hazards are not significant predictors of quality of life.

Older adults who perceived their neighbourhoods as having high levels of places for walking, aesthetics, service accessibility, and criminal safety were more likely to have better quality of life than those who perceived their neighbourhoods as having lower rates in physical environments, 1.34, 1.40, 1.75, and 1.80 times, respectively. Further, older adults who perceived their neighbourhoods as having higher in social cohesion, social support and social trust were more likely to report better quality of life than those who perceived their neighbourhoods as having lower rates in social environments, 1.47, 1.52, and 2.46 times, respectively.

## 4. Discussion

The study found among a large sample of older adults in Thailand significant associations between perceived age-friendly environments, in particular physical, security and social environments, and quality of life. Overall, older adults who perceived a high level of age-friendliness in their neighbourhoods were 3.79 times more likely to have better quality of life than older adults who perceived low levels of age-friendliness in their neighbourhood. Among age-friendly environment predictors, neighbourhood social trust appeared to have the greatest positive impact on quality of life among this Thai older adult population. The strongest predictor of quality of life was neighbourhood social trust, followed by neighbourhood criminal safety, neighbourhood service accessibility, neighbourhood social support, neighbourhood social cohesion, neighbourhood aesthetics, and walkable neighbourhood.

In agreement with previous studies [9,10,11], this study found a significant positive relationship between neighbourhood social trust, social support and social cohesion and quality of life among the older population. Another study from the Netherlands described that social cohesion and social support among neighbours may increase levels of well-being in older adults by way of providing affective support, increasing self-esteem and enhancing mutual respect [8]. In a study from Canada it was found that only a sense of trust in people within one’s family was found to be associated with people’s well-being, whereas a sense of trust in neighbours was not [14]. However, the participants in that study were 15 years of age and older, and the study was not adjusted by the number of years participants had been living in their community. In agreement with our study findings was a study in a developing country, Colombia, that found that older adults with the highest perception of trust in their neighbourhood had better mental health-related quality of life (HRQOL) compared to their counterparts with the lowest perception of trust [14]. The researchers of that study [14] believed that higher levels of “trust in others” enabled them to reduce negative emotions, stress levels, and anxiety, and they were more likely to increase feelings of security and self-esteem, leading to better mental health as well as initiating collective and coordinated actions that might directly promote health by limiting or delaying the onset of disease and functional disability [15].

Regarding physical or built environments, the significant relationship of neighbourhood accessibility, neighbourhood aesthetics and places for walking in neighbourhood and quality of life in this study was also found in several previous studies [5,15,16,17,18,19,20,21,22,29,31]. For example, in a study on neighbourhood aesthetics, previous research from Berlin addressed that residents living in a more aesthetic neighbourhood, such as a neighbourhood with more vegetation view, have significantly better conditions of mental health such as lower stress and higher life satisfaction [16]. In agreement with the Berlin research, previous research from the United States also documented the important role of natural environments, such as air, water, open space, and cleanliness around neighbourhoods on quality of life among Vermont residents [17]. Moreover, a study in the Netherlands also indicated that quality and quantity of greenspace were positively related with self-rated health among Dutch residents in an urban neighbourhood [32]. It is reasonable to consider that residents living near greenspace or natural environment filled with recreational resources would spend more time on physical activity, and can, thus, improve functional status and well-being [33]. Another study from the United States pointed out that pleasantness surrounding people’s homes provided the older people with easy and attractive routes with which to remain active and enjoy a good quality of life [18]. Recent research from China and Brazil also support that aesthetic neighbourhoods are associated with improved physical health-related quality of life among Chinese adults aged 30–55 years [11], and are associated with better mental health-related quality of life among the Brazilian population [12]. Additionally, findings from a mixed method study indicate that a pleasant physical environment surrounding a social care institution plays a significant role in enhancing quality of life amongst older people in Malaysia [13]. In respect to walkable neighbourhoods, a review from the United States [19] documented that green environment in respect to walkability was strongly related to the health and well-being of older adults. In a study from Indonesia, a significant relationship between public, open space-related walking and the physical quality of life among residents in a low-income community was found [20]. However, some studies from the United States found no association between neighbourhood walkability and health-related quality of life among adults in higher income neighbourhoods [34]. In case of neighbourhood service accessibility, a study from the United States found a significant relationship between well-being and neighbourhood accessibility among US elderly in ten major cities [21]. Yet another study from the UK [22] indicated that accessibility was a main predictor of quality of life among British elderly aged 65 years and older, due to the easy access to local facilities and transport bringing about independence of the elderly. Overall, these findings seem to support the importance of mobility and accessibility with regard to quality of life in older adults. We did not find an association between neighbourhood street connectivity and quality of life among Thai older persons. This may be because the Thai older population is not accustomed to engaging in physical activity such as walking in the streets or through a block network. Instead, they may have physical activity such as walking or jogging in a public park or recreational area.

Concerning neighbourhood crime, in agreement with our results, a prior study from Brazil found a significant association between neighbourhood criminal safety and physical as well as mental health-related quality of life [12]. It is possible that the fear of crime may negatively impact mental health, such as incite depression [35]. In addition, fear of crime may have an impact on social well-being. For example, crime reduced trust and cohesion within communities and caused individual withdrawal, which then impacted negatively on well-being or quality of life [23]. Unlike previous studies [23,24,25,26], this study did not find an association between traffic safety and the quality of life among Thai older adults. It is possible that the NEWS-A sub-scale measuring traffic hazards did not fully capture the Thai context of traffic safety. More research, particularly on the dimension of age-friendly traffic safety is required.

The results of this study can assist planners in the design of an age-friendly city and the management of age-friendly environments so as to promote high quality of life among the older population. A combination of social and physical or built environments in a neighbourhood, such as social trust, social cohesion, social support, accessibility, aesthetics, walking trails, and safe environments will contribute to healthier and more livable aging communities. Other age-friendly environments, such as quality and quantity of health care service, and economic development are also essential the effort to build healthier and more livable aging communities.

There are some limitations to this study. First, the nature of the cross-sectional design of the study cannot confirm the causal relationships between quality of life and the predictors. Second, age-friendly environments are comprised of many dimensions, such as housing, transportation, civic participation and employment, respect and social inclusion, and communication and information, some of which the present study did not include, and should be included in future studies. Third, the measurement of age-friendly environments in this study relied on perceived rather than objective measures of the environments. However, the strength of the present study lies in the large representative sample of older adults in Thailand utilized, linking age-friendly environments to quality of life in preparation for an aging society.

## 5. Conclusions

Moving toward an age-friendly environment is one of the most important challenges in responding to demographic transition. The present study significantly supported the important roles of age-friendly environments in impacting quality of life among the older population in Thailand. In order to build an age-friendly community, the improvement of physical or built environments, social environments, and security within that community is a challenging issue that must be considered.

## Figures and Tables

**Table 1:** Sample characteristics (*n* = 4183).

Variable	*n*	Percent
Age in years		
60–69	2272	54.3
70–79	1378	32.9
80 or more	533	12.7
Gender		
Male	1482	35.4
Female	2701	64.6
Educational level		
No education	344	8.2
Completed elementary school	3093	73.9
Completed middle/high school/associate degree	505	12.1
Completed bachelor degree or higher	241	5.8
Marital status		
Single	278	6.6
Married	2446	58.5
Separated/divorced/widowed	1459	34.9
Income level per month		
Less than 5000 Baht ^1^	3122	74.6
5001–10,000 Baht	567	13.6
10,001–20,000 Baht	308	7.4
20,001 Baht and higher	186	4.4
Residence		
Rural	1906	45.6
Urban	1884	45.0
Metropolitan (Bangkok)	393	9.4
Living arrangement		
Living alone	326	7.8
Living with others	3857	92.2
Number of years living in this community		
1–10 years	364	8.7
More than 10 years	3819	91.3
Health problem		
No	1006	24.0
Yes	3177	76.0

^1^ One US $ = 35 Baht.

**Table 2:** Descriptive statistics of age-friendly environments and quality of life.

Variable	*n*	Percent
**Neighbourhood built environments**		
Neighbourhood service accessibility		
High	2642	63.2
Low	1541	36.8
(Min–Maximum = 4–16, cut point = 10) ^1^		
Neighbourhood street connectivity		
High	2248	53.7
Low	1935	46.3
(Min–Maximum = 3–12, cut point = 8)		
Places for walking in neighbourhood		
High	2292	54.8
Low	1891	45.2
(Min–Maximum = 6–24, cut point = 16)		
Neighbourhood aesthetics		
High	2586	61.8
Low	1597	38.2
(Min–Maximum = 4–16, cut point = 11)		
**Neighbourhood safety**		
Neighbourhood crime		
High	2291	54.8
Low	1892	45.2
(Min–Maximum = 3–12, cut point = 4)		
Neighbourhood traffic hazards		
High	2330	55.7
Low	1853	44.3
(Min–Maximum = 3–12, cut point = 6)		
**Neighbourhood social environments**		
Neighbourhood social trust		
High	2326	55.6
Low	1857	44.4
(Min–Maximum = 3–12, cut point = 10)		
Neighbourhood social cohesion		
High	3151	75.3
Low	1032	24.7
(Min–Maximum = 3–12, cut point = 9)		
Neighbourhood social support		
High	3475	83.1
Low	708	16.9
(Min–Maximum = 3–12, cut point = 9)		
Total neighbourhood age-friendly environments		
High	2151	51.4
Low	2032	48.6
(Min–Maximum = 17–98, cut point = 63)		
Quality of life		
Poor/normal	2684	64.2
Good	1499	35.8
(Min–Maximum = 30–130, the cut point = 96)		

^1^ Minimum and maximum scores on subscale, median cut point value.

**Table 3:** Factors associated with quality of life among Thai older adults using χ^2^ statistics.

Items	Level of Quality of Life *			
	Poor/Normal		Good	
	*n*	%	*n*	%
Gender				
Male	955	64.4%	527	35.6%
Female	1729	64.0%	972	36.0%
Educational level ***				
No education	296	86.0%	48	14.0%
Completed elementary school	2036	65.8%	1057	34.2%
Completed middle/high school/associate degree	255	50.5%	250	49.5%
Completed bachelor degree or higher	97	40.2%	144	59.8%
Marital status ***				
Single	192	69.1%	86	30.9%
Married	1503	61.4%	943	38.6%
Separated/divorced/widowed	989	67.8%	470	32.2%
Income level per month ***				
Less than 5000 Baht	2120	67.9%	1002	32.1%
5001–10,000 Baht	330	58.2%	237	41.8%
10,001–20,000 Baht	160	51.9%	148	48.1%
20,001 Baht and higher	74	39.8%	112	60.2%
Age level ***				
60–69 years	1377	60.6%	895	39.4%
70–79 years	898	65.2%	480	34.8%
80 years and higher	409	76.7%	124	23.3%
Residence **				
Rural	1276	66.9%	630	33.1%
Urban	1168	62.0%	716	38.0%
Metropolitan (Bangkok)	240	61.1%	153	38.9%
Health problem ***				
No	557	55.4%	449	44.6%
Yes	2127	66.9%	1050	33.1%
Living arrangement				
Living alone	222	68.1%	104	31.9%
Living with others	2462	63.8%	1395	36.2%
Number of years living in this community ***				
1–10 years	267	73.4%	97	26.6%
More than 10 years	2417	63.3%	1402	36.7%
**Neighbourhood built environments**				
Living in neighbourhood with service accessibility ***				
High	1494	56.5%	1148	43.5%
Low	1190	77.2%	351	22.8%
Living in neighbourhood with street connectivity ***				
High	1332	59.3%	916	40.7%
Low	1352	69.9%	583	30.1%
Living in neighbourhood with places for walking ***				
High	1296	56.5%	996	43.5%
Low	1388	73.4%	503	26.6%
Neighbourhood aesthetics ***				
High	1490	57.6%	1096	42.4%
Low	1194	74.8%	403	25.2%
**Neighbourhood securities**				
Living in neighbourhood with crime ***				
High	1629	71.1%	662	28.9%
Low	1055	55.8%	837	44.2%
Living in neighbourhood with traffic hazards **				
High	1542	66.2%	788	33.8%
Low	1142	61.6%	711	38.4%
**Neighbourhood social environments**				
Neighbourhood social trust ***				
High	1211	52.1%	1115	47.9%
Low	1473	79.3%	384	20.7%
Neighbourhood social cohesion ***				
High	1859	59.0%	1292	41.0%
Low	825	79.9%	207	20.1%
Neighbourhood social support ***				
High	2113	60.8%	1362	39.2%
Low	571	80.6%	137	19.4%

* Poor or normal quality of life was defined as the total score of quality of life below 96, whereas good quality of life was defined as the total score of quality of life equal to or above 96; ** *p* < 0.01; *** *p* < 0.001.

**Table 4:** Total age-friendly environments (physical, security, and social environments), socio-demographic factors and health conditions as predictors of quality of life among Thai older adults.

Predictors	Adjusted Odds Ratio	95% CI	
		Lower	Upper
Perceived age-friendly environments ***			
High	3.793	3.296	4.365
Low (Reference)			
Educational level ***			
No education (Reference)			
Completed elementary school	2.382	1.715	3.307
Completed middle/high school/associate degree	4.188	2.862	6.128
Completed bachelor degree or higher	4.945	3.076	7.949
Marital status *			
Single (Reference)			
Married	1.388	1.039	1.855
Separated/divorced/widowed	1.290	.953	1.747
Income level per month **			
Less than 5000 Baht (Reference)			
5001–10,000 Baht	1.348	1.102	1.649
10,001–20,000 Baht	1.382	1.046	1.826
20,001 Baht and higher	1.572	1.065	2.322
Age level **			
60–69 years	1.456	1.145	1.852
70–79 years	1.431	1.117	1.834
80 years and higher (Reference)			
Residence			
Rural (Reference)			
Urban	1.165	1.000	1.357
Metropolitan (Bangkok)	1.114	.863	1.437
Health problem ***			
No	1.682	1.434	1.974
Yes (Reference)			
Number of years living in the community ***			
1–10 years (Reference)			
More than 10 years	1.649	1.265	2.149

CI = Confidence Interval; * *p* < 0.05; ** *p* < 0.01; *** *p* < 0.001.

**Table 5:** Different dimensions of age-friendly environments as predictors of quality of life among Thai older adults.

Predictors	Adjusted Odds Ratio ^1^	95% CI	
		Lower	Upper
**Neighbourhood built environments**			
Neighbourhood service accessibility ***			
High	1.751	1.475	2.080
Low (Reference)			
Neighbourhood street connectivity			
High	0.861	0.729	1.016
Low (Reference)			
Places for walking in neighbourhood ***			
High	1.337	1.136	1.574
Low (Reference)			
Neighbourhood aesthetics ***			
High	1.403	1.194	1.648
Low (Reference)			
**Neighbourhood securities**			
Neighbourhood crime ***			
Low	1.802	1.532	2.120
High (Reference)			
Neighbourhood traffic hazards			
Low	1.023	0.867	1.206
High (Reference)			
**Neighbourhood social environments**			
Neighbourhood social trust ***			
High	2.463	2.104	2.885
Low (Reference)			
Neighbourhood social cohesion ***			
High	1.470	1.196	1.807
Low (Reference)			
Neighbourhood social support **			
High	1.518	1.194	1.929
Low (Reference)			

^1^ Adjusted for education, marital status, income, age, residence, health problems, and number of years living in the community; CI = Confidence Interval; ** *p* < 0.01; *** *p* < 0.001.
